# Time to first dose of measles-containing vaccine and associated factors among infants in Ethiopia: a survival analysis from performance monitoring for action data

**DOI:** 10.3389/fpubh.2025.1521602

**Published:** 2025-10-16

**Authors:** Eyob Tilahun Abeje, Ermias Bekele Enyew, Chala Daba, Lakew Asmare, Fekade Demeke Bayou, Mastewal Arefaynie, Anissa Mohammed, Abiyu Abadi Tareke, Awoke Keleb, Natnael Kebede, Yawkal Tsega, Abel Endawkie, Shimels Derso Kebede, Kaleab Mesfin Abera

**Affiliations:** ^1^Department of Epidemiology and Biostatistics, School of Public Health, College of Health Sciences, Wollo University, Dessie, Ethiopia; ^2^Department of Health Informatics, School of Public Health, College of Medicine and Health Sciences, Wollo University, Dessie, Ethiopia; ^3^Department of Environmental Health College of Medicine and Health Sciences, Wollo University, Dessie, Ethiopia; ^4^Department of Epidemiology and Biostatistics, Institute of Public Health, College of Medicine and Health Science, University of Gondar, Gondar, Ethiopia; ^5^Department of Reproductive and Family Health, School of Public Health, College of Medicine and Health Sciences, Wollo University, Dessie, Ethiopia; ^6^Nossal Institute for Global Health, Melbourne School of Population and Global Health, University of Melbourne, Parkville, VIC, Australia; ^7^Department of Health Promotion, School of Public Health, College of Medicine and Health Sciences, Wollo University, Dessie, Ethiopia; ^8^Department of Health System and Management, School of Public Health, College of Medicine and Health Sciences, Wollo University, Dessie, Ethiopia

**Keywords:** measles-containing vaccine, timeliness of measles first dose, child health, measles immunization, survey design survival analysis, MCV1

## Abstract

**Introduction:**

The measles-containing vaccine (MCV) is a live attenuated vaccine that helps to develop lifelong immunity, and it prevents measles outbreak when administered at the right time in measles-endemic areas. Many infants received the initial dose of the measles vaccine later than the ideal time frame, and significant others missed the vaccination, causing a recurrent measles outbreak in Ethiopia. This study assessed the time to the first dose of a measles-containing vaccine and associated factors among infants in Ethiopia.

**Methods:**

A cohort of 1,770 mother–infant pairs was analysed using data from the performance monitoring for action Ethiopia dataset. Cohort-2 Ethiopian data set was collected in Addis Ababa, Amhara, Oromia and SNNP regions between between November 2021 and August 2023. The key independent variables were socio-demographic characteristics, maternal health service utilization, and pregnancy intention. Multiple imputation was used to handle missing data. Survival analysis was conducted using R programming language version 4.4.1. Multicollinearity was assessed using Generalized variance inflation factors (GVIF), and model fit was evaluated using concordance index and overall model significance.

**Results:**

Among 1,770 infants followed, only 27% were vaccinated timely, within 9–10 months of age (survival probability = 0.73), and 53.4% had not yet received MCV1 at 12 months of age. The hazard of receiving the first dose of measles vaccine (MCV1) was 35% lower among infants from pregnancies that were not desired at all (AHR = 0.65, 95% CI: 0.46-0.93) and 21% lower among those infants from pregnancies that were initially undesired but later became wanted (AHR = 0.79, 95% CI: 0.65–0.96), compared to infants from pregnancies that were desired from the beginning.

**Conclusion:**

Despite progress in the uptake of the first dose of measles vaccine, timely vaccination in Ethiopia is still low, and many infants in Ethiopia miss the immunization. Institutional delivery, maternal intention regarding pregnancy, religion, and wealth quantile were key predictors of the timeliness of the first dose of measles vaccine. Interventions encouraging institutional deliveries, supporting unintended pregnancy, working with religious leaders, and conducting continuous outreach to immunization services are necessary to improve the timely uptake of the first dose of measles vaccine.

## Introduction

Expanded Programme on Immunization (EPI) was launched in 1974 to provide children with access to life-saving vaccines around the globe. Since then, a national immunization program has started in every country in the world and has averted the deaths of 154 million children worldwide until 2024. Now, EPI has grown into what is commonly recognized as the EPI (1, 2).

Measles is the cause of morbidity and mortality for millions, despite the efforts taken, including measles vaccinations, surveillance, and outbreak investigation. The estimated number of measles cases increased by 20% worldwide, from 8,645,000 to 10,341,000, and the estimated number of measles deaths decreased by 8%, from 116,800 to 107,500, in 2023 compared to 2022. The decrease in measles deaths compared to cases is due to the increased number of cases occurring in countries with a lower risk of death (3). In Ethiopia, there were 112 measles outbreaks in 108 districts and 1,503 confirmed measles cases in 2020 (4). The first dose of measles containing-vaccine (MCV) is administered when infants reach 9 months of age in measles-endemic areas, and at 12–15 months of age in areas where measles is not common. The second dose is delivered at 15–18 months of age in endemic areas and at ages 4–6 years in non-endemic areas, such as the United States. Measles-containing vaccine (MCV) protects a vaccinated child from acquiring the measles virus and protects the community through the development of herd immunity, which is achieved when 95% of the community is vaccinated. In Ethiopia, measles-containing vaccine one (MCV1) is administered starting at 9 months, and measles-containing vaccine two (MCV2) is administered at 15 months of age (5–9).

A systematic analysis of the Global Burden of Diseases, Injuries, and Risk Factors Study (GBD) on National Trends in Routine Childhood Vaccination showed that the long-term improvement in immunization reduced between 2010 and 2019, exacerbated by COVID-19, and was unable to reach the pre-COVID state in 2023 (2). Immunization Agenda 2030 (IA2030) is set to achieve 90% coverage for DTP3, PCV3, MCV2, and HPV vaccines. Forecasts to 2030 showed that only DPT3 may reach IA2030 (2). Vaccine hesitancy in Africa is a drawback to achieving IA2030 (2, 10). The WHO–UNICEF estimates MCV1 and MCV2 coverage for the African Region were 69 and 45% in 2022, respectively. In addition, Ethiopia is one of the eight countries in the globe that comprise 50% of zero-dose children (2, 11).

The worldwide MCV1 coverage was 72% in 2000 and increased to 86% in 2019, as reported by WHO. Nevertheless, it was found that MCV1 coverage decreased to 81% in the 2022 report, after Coronavirus Disease 2019 (COVID-19); it is the lowest achievement of MCV1. It rose and reached 83% between 2022 and 2023, but is still short of achieving the WHO target of 95% MCV1 immunization coverage. As a result of the COVID-19 pandemic, there was an 18% increase in measles cases worldwide as well (5, 6, 11). Sub-Saharan Africa consists of over 52.6% of zero-dose children. Ethiopia holds the third spot with 1.7 million MCV unvaccinated infants, next to Nigeria (3 million) and the Democratic Republic of the Congo (1.8 million) among 10 countries that contributed to 52% MCV unvaccinated children under 5 years of age (2, 5). According to the 2016 Ethiopian Demographic and Health Survey (EDHS) report, MCV1 coverage was 54.3% whereas it was 59% in the 2019 mini EDHS (12–14). Many infants are at significant risk of acquiring the measles virus since they did not take the vaccine, which causes measles outbreaks. It was found that only 26.4% of children received the measles vaccine on time, leading to delayed immunity and higher vulnerability to measles (9).

Missed and untimely vaccinations compromise the child’s development of permanent immunity and ultimately lead to the development of measles outbreaks. The measles outbreak is impacting a health system that has weekend by COVID-19 pandemic and remains highly unstable due to the conflict in Ethiopia. In low-income countries, such as sub-Saharan African countries, the burden of measles continues to affect the health system as a result of low MCV coverage and poor outbreak management. Measles infection has been a persistent problem, with repeated outbreaks reported in several regions, due to untimely and missed measles vaccinations in Ethiopia. The measles virus is highly contagious, and children who do not receive MCV1 at the appropriate age are at greater risk of infection, leading to increased severe complications, such as pneumonia, encephalitis, and death, mainly among children under 5 years of age (15–20).

It has been found that several factors are associated with delayed or missed measles vaccination. Socioeconomic status, maternal education, access to immunization services such as vaccine stockouts, long distances to health facilities, rural residence, and logistical challenges such as vaccine availability, and lack of awareness about the benefit of vaccinations and vaccination schedules were found to be associated factors for timely MCV1 uptake (12, 21–27).

To ensure adequate protection, the WHO and Ethiopian Ministry of Health guidelines state that MCV1 should be administered at 9 months, followed by subsequent vaccination at 15 months of age. Strengthening routine immunization services, such as outreach immunization services for hard-to-reach areas and increasing community engagement, has paramount importance to promote timely vaccination, as stated by global and national guidelines (20, 28, 29). Sustainable Development Goal 3 (SDGs) stated that ensuring healthy lives and promoting wellbeing for all ages, targets reducing child mortality through universal immunization coverage. Expanding measles immunization is crucial to meet the SDG 3.2 goal of ending preventable deaths of newborns and children under 5 years of age (30). So far, these strategies have brought about changes in MCV coverage, even though it is not enough to achieve herd immunity and prevent recurrent measles outbreaks in Ethiopia. However, ongoing conflicts in Oromia, Amhara, and the Tigray region affects the coordination of different stakeholder immunization services in Ethiopia (9, 12, 15, 21, 31, 32).

This study assessed the timing of MCV1 vaccination and its associated factors among infants in Ethiopia using data from the Performance Monitoring for Action (PMA). This study gives valuable evidence for policymakers and health professionals who are engaged in interventions on timely vaccination and coverage, ultimately reducing the burden of the measles outbreak in Ethiopia through identifying predictors for timely immunization of MCV1 to address the ongoing challenges of childhood vaccination in Ethiopia and ensuring that all children receive their vaccinations on time.

## Methods

### Study area and period

The Performance Monitoring for Action (PMA) survey is a major source of data on reproductive, maternal, and newborn health (RMNH) for decision-making. It has been conducted in many countries, such as Ethiopia. The project conducts cross-sectional and cohort surveys to fill data gaps not addressed by other major surveys, such as RMNH care services and the factors influencing their delivery across Amhara, Oromia, SNNP, and Addis Ababa regions. PMA Cohort-2 data were collected from November 2021 to August 2023, which consisted of baseline data on socio-demographic characteristics, pregnancy intentions, and antenatal care services from pregnant and 5–9 weeks postpartum. Eligible women were followed at 6 weeks, 6 months, and 1 year after childbirth. This study includes those mother-infant pairs who participated and completed the 1-year follow-up period (33–38).

### Study design

A cohort study was conducted. At baseline, a cross-sectional design was conducted to enroll eligible women and collect initial data.

#### Population

##### Source population

The source population includes all infant-mother pairs residing in the selected regions of Ethiopia (2021–2023).

##### Study population

The study population consisted of all infant-mother pairs residing in the selected regions of Ethiopia with infants who were either 9 weeks of age by November 2021 or born between November 2021 and October 2022. All infants were required to survive to at least 9 months of age.

#### Eligibility criteria

##### Inclusion criteria

The inclusion criteria consisted of all infants aged 9 months and older from cohort 2 of the PMA data (2021–2023).

##### Exclusion criteria

Children who received MCV1 before 9 months (270 days) of age.

#### Sample size determination

##### Sample size determination PMA

PMA Ethiopia determined the sample size using data from previous PMA2020 surveys to estimate the modern contraceptive prevalence rate, account for the design effect, and anticipate non-response. A total of 217 enumeration areas (EAs) were selected to achieve a 5% margin of error for modern contraceptive prevalence rate estimates in each panel region, with an additional 81 EAs included for non-panel regions. Based on fertility rates, approximately 2,800 women were expected to enroll in the panel for cohort-1. The cohort-2 was updated from this cohort-1 as Tigray, Afar, and the eight EAs in SNNP were removed in cohort-2. Finally, the final EA sample size for cohort-2 was 162 EAs (38).

##### The sample size included in this study

PMA cohort 2 included 10,389 women in the baseline survey. Among these, only 2,297 were eligible for the follow-up survey, which comprised women who were pregnant (1,796), 0–4 weeks postpartum (228), and 5–9 weeks postpartum (273). A total of 8,092 women were not enrolled into the cohort-2 as they were neither pregnant nor postpartum at baseline. Finally, 1,990 were followed up on until the 1-year follow-up period, and 1,859 consented and completed the 1-year follow-up survey. After excluding infants who received MCV1 before 9 months of age, 1,770 mother-infant pairs were included in the analysis.

#### Sampling technique and procedure

A multistage stratified cluster sampling method was utilized for PMA data. The enumeration areas (EAs) were chosen with probability proportional to size within the strata. In Amhara, Oromia, and SNNP, the strata were defined by urban/rural residence, while no strata applied to Addis ababa. Within the panel regions, census of households was conducted, all women aged 15–49 who were regular household members were identified. Eligible participants for follow-up were those who were pregnant or had given birth in the past 9 weeks (38).

#### Study variables

##### Dependent variables

Time to first dose of MCV (vaccinated or event = 1, censor = 0).

##### Independent variables

*Socio-demographic characteristics*: religion, women’s age group, strata, education status of women, residence, wealth quintile, living together after marriage, and marital status.

*Reproductive variables*: desired pregnancy, parity, delivery place, and PNC vaccination counseling.

#### Operational definition

*Survival time*: Time from 9 months (269 days) to 1-year follow-up. The vaccination date was obtained from the child’s immunization card. For those who were censored, survival time was calculated from the date of the last follow-up interview. For individuals without an immunization card, survival time was estimated using multiple imputation techniques (34, 35). Time zero was defined at 269 days of age, and the event was considered to occur at 270 days of age (9 months) to assess timely vaccination during the follow-up period. Children who received MCV1 before 270 days of age were excluded from the analysis. This prevents the misclassification of early vaccinations, which are affected by maternal antibodies, as a good outcome.

*Event*: When the infant took the MCV1 vaccine, starting from 270 days till the follow-up period ends.

*Timely MCV1 vaccination*: When the infant received MCV1 between 9–10 completed months of age (4, 9, 39).

### Data extraction tools and procedure

During the census, enumerators screened women to identify those who were pregnant or had given birth within the past 9 weeks. During the initial interview, data were collected on various socio-demographic characteristics. Women who consented to participate were enrolled in the follow-up survey and asked about maternal and child health until 1-year follow-up. Data were collected at each visit only for children who were still alive. Since the study focused on time to MCV1, only women whose children were alive and who completed the 1-year follow-up cohort were included in the analysis (38).

### Data quality management

To ensure data quality, data collectors and supervisors received training and ongoing monitoring throughout the data collection process. Daily data checks were conducted, and the field teams received feedback to address any discrepancies. Additionally, data validation rules were integrated into the ODK system to minimize entry errors and enhance the reliability of the collected data (38).

### Data processing and analysis

Data were cleaned, processed, and analyzed using R statistical software. Descriptive statistics were calculated to summarize key indicators, and multiple imputations were applied to address missing values in the variables. Sensitivity analysis was conducted, and the results were pooled using Rubin’s rule. Survival analysis was conducted using the survey package, which accounts for complex survey design in R (version 4.4.1) to account for the clustering, strata, and weighting effects inherent in the survey design. The proportional hazards assumption held for all variables. Multicollinearity was checked. Variable selection was conducted based on the likelihood significance test. Model fitness was assessed using the concordance index value (concordance index = 0.74), and the overall model significance test.

### Ethical approval

The dataset was obtained from the PMA website via email after presenting the study’s objective and overall purpose.

## Results

### Socio-demographic variables

Out of 1,770 participants, the largest proportion, 419 (23.67%), was from Oromia-2, followed by SNNP-2 with 362 (20.45%), and the least from Amhara-1 with 129 (7.29%). Regarding residence, 1,023 (57.80%) of respondents were from rural areas. The lowest, lower, middle, higher, and highest wealth quintiles accounted for 282 (15.93%), 271 (15.31%), 284 (16.05%), 339 (19.15%), and 594 (33.56%) participants, respectively (Table 1).

**Table 1:** Socio-demographic variables of time to MCV1 from PMA data.

Variable	Percent of missingness	Category	Frequency (percentage)	
			Before the imputation of missing data	After the imputation of missing data
Religion	0%	1. Orthodox	671 (37.91)	671 (37.91)
		2. Protestant	565 (31.92)	565 (31.92)
		3. Moslem/Muslim	512 (28.93)	512 (28.93)
		4. other	22 (1.24)	22 (1.24)
		Missing	0 (0.00)	0 (0.00)
Women age group	0%	<25	584 (32.99)	584 (32.99)
		25–34	908 (51.30)	908 (51.30)
		35+	278 (15.71)	278 (15.71)
		Missing	0 (0.00)	0 (0.00)
Strata	0%	10. Addis	246 (13.90)	246 (13.90)
		3. Amhara-1	129 (7.29)	129 (7.29)
		3. Amhara-2	242 (13.67)	242 (13.67)
		4. Oromiya-1	215 (12.15)	215 (12.15)
		4. Oromiya-2	419 (23.67)	419 (23.67)
		7. SNNP-1	157 (8.87)	157 (8.87)
		7. SNNP-2	362 (20.45)	362 (20.45)
		Missing	0 (0.00)	0 (0.00)
Educational level of the mother	0%	0. Never attended	475 (26.84)	475 (26.84)
		1. Primary	769 (43.45)	769 (43.45)
		2. Secondary	292 (16.50)	292 (16.50)
		3. Technical and vocational	84 (4.75)	84 (4.75)
		4. Higher	150 (8.47)	150 (8.47)
		Missing	0 (0.00)	0 (0.00)
Residence	0%	1. Urban	747 (42.20)	747 (42.20)
		2. Rural	1,023 (57.80)	1,023 (57.80)
		Missing	0 (0.00)	0 (0.00)
Wealth quintile	0%	1. Lowest quintile	282 (15.93)	282 (15.93)
		2. Lower quintile	271 (15.31)	271 (15.31)
		3. Middle quintile	284 (16.05)	284 (16.05)
		4. Higher quintile	339 (19.15)	339 (19.15)
		5. Highest quintile	594 (33.56)	594 (33.56)
		Missing	0 (0.00)	0 (0.00)
Living together after marriage	1.02%	1. >5	1,031 (58.25)	1,040 (58.76)
		1_2	490 (27.68)	494 (27.91)
		3–5	231 (13.05)	236 (13.33)
		Missing	18 (1.02)	0 (0.00)
Marital status of the mother	0%	1. Married	1,662 (93.90)	1,662 (93.90)
		2. Living with a partner	69 (3.90)	69 (3.90)
		3. Divorced/separated	18 (1.02)	18 (1.02)
		5. Never married	18 (1.02)	18 (1.02)
		4. Widow	3 (0.17)	3 (0.17)
		Missing	0 (0.00)	0 (0.00)

### Maternal and reproductive health factors

Desired pregnancy comprises 1,176 (66.44%) of the participants. Home delivery was accountable for 526 (29.72%), while institutional deliveries, government hospitals (28.14%), government health centers (38.02%), and other institutional deliveries (4.12%) comprise the rest (Table 2).

**Table 2:** Maternal and reproductive health factors.

Variable	Percent of missingness	Category	Frequency (percentage)	
			Before the imputation of missing data	Before the imputation of missing data
Last pregnancy desired/wanted	0%	1. Then	1,176 (66.44)	1,176 (66.44)
		2. Later	510 (28.81)	510 (28.81)
		3. Not at all	84 (4.75)	84 (4.75)
		Missing	0 (0.00)	0 (0.00)
Status	11.02%	0	505 (28.53)	601 (33.95)
		1	1,070 (60.45)	1,169 (66.05)
		Missing	195 (11.02)	0 (0.00)
Parity	6.67%	≤2	1,008 (56.95)	1,116 (63.05)
		>4	286 (16.16)	287 (16.21)
		3–4	358 (20.23)	367 (20.73)
		Missing	118 (6.67)	0 (0.00)
Delivery	4.58%	1. Home	512 (28.93)	526 (29.72)
		11. Government hospital	462 (26.10)	498 (28.14)
		12. Government health center	648 (36.61)	673 (38.02)
		96. Other	67 (3.79)	73 (4.12)
		Missing	81 (4.58)	0 (0.00)
PNC vaccination counseling	71.47%	0. No	214 (12.09)	791 (44.69)
		1. Yes	291 (16.44)	979 (55.31)
		Missing	1,265 (71.47)	0 (0.00)

Other, other private medical sector, NGO/Faith-based health facility, and private hospital/clinic.

### Measles first dose

**Figure 1:**
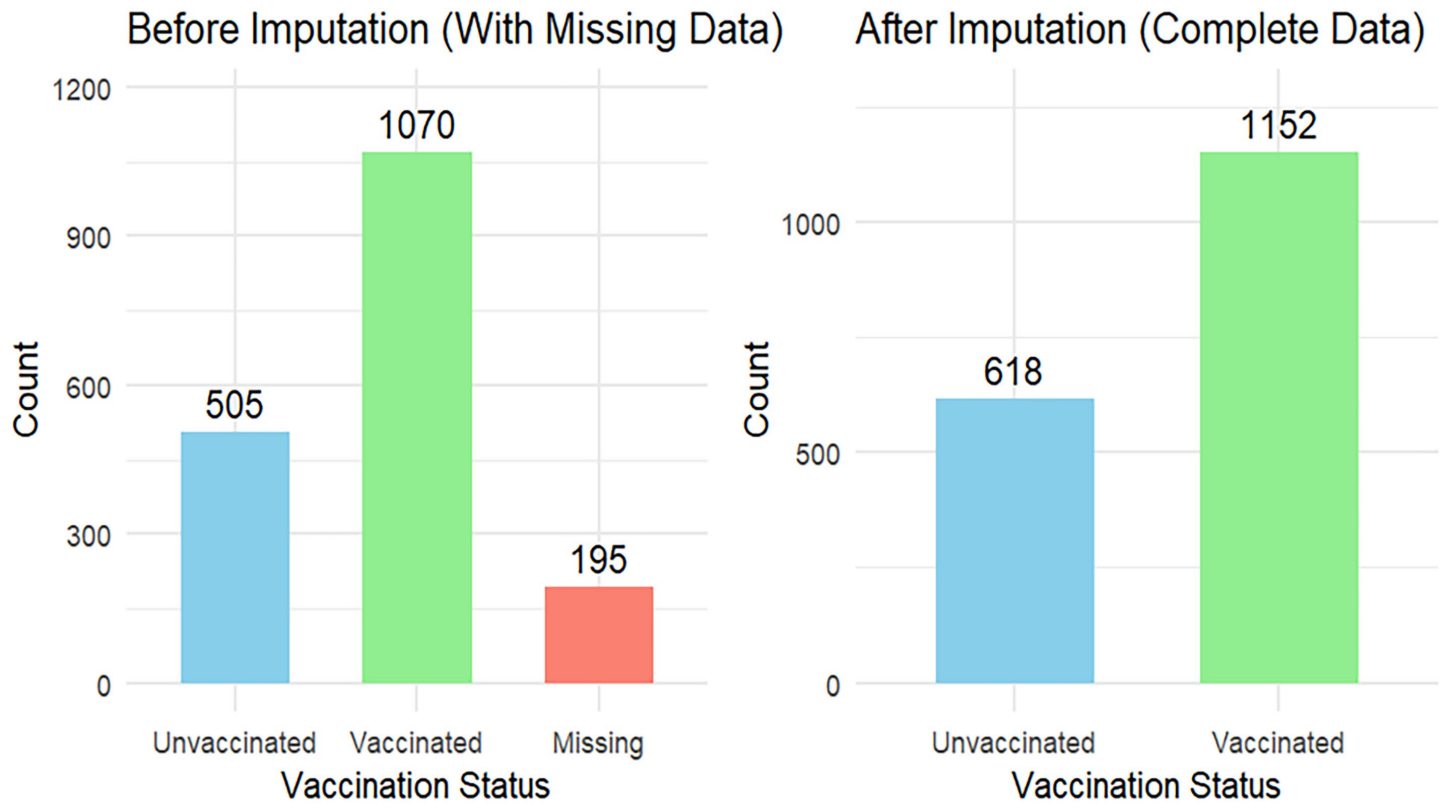
Survival status distribution of measles first dose.

Among 1,770 participants, 618 (65.08%) developed an event of interest with a maximum follow-up time of 146 days. The incidence density was 12 per 1,000 person-days of follow-up (Figure 1).

### Survival probability

**Figure 2:**
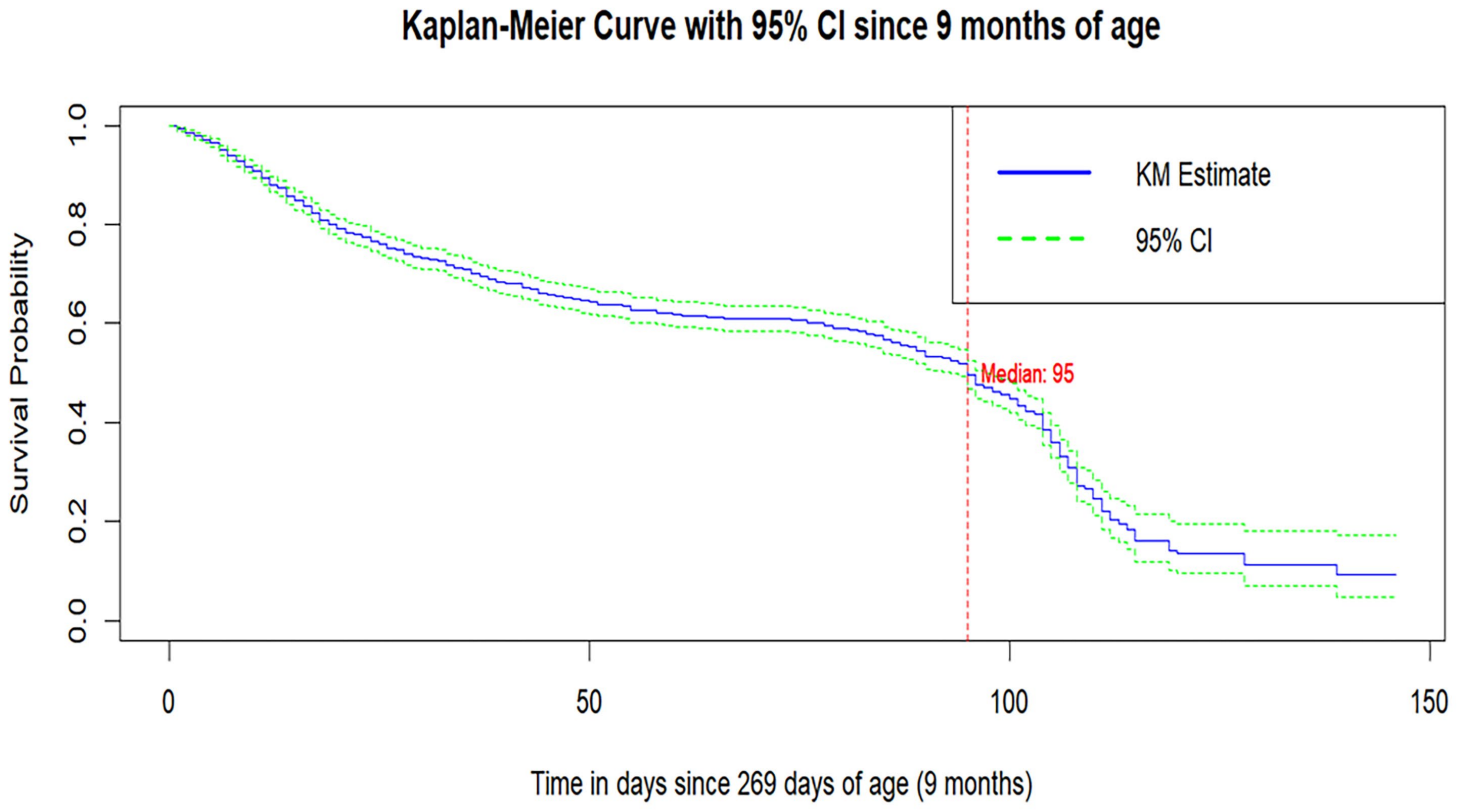
Baseline survival curves of time to MCV1.

The median survival time to MCV1 vaccination was 95 days after the child turned 9 months old. The survival probability of MCV1 at 10 months of age since the infant completed 9 months of age was 0.73 (95%CI: 0.708,0.75), which indicates that 27% of infants took MCV1 at 10 months of age. The survival probability of MCV1 at 1 year of age was 0.534 (95%CI: 0.507, 0.562), which indicates that 46.6% of them received MCV1 on their first birthday (Figure 2).

### Survival probabilities for independent variables

**Figure 3:**
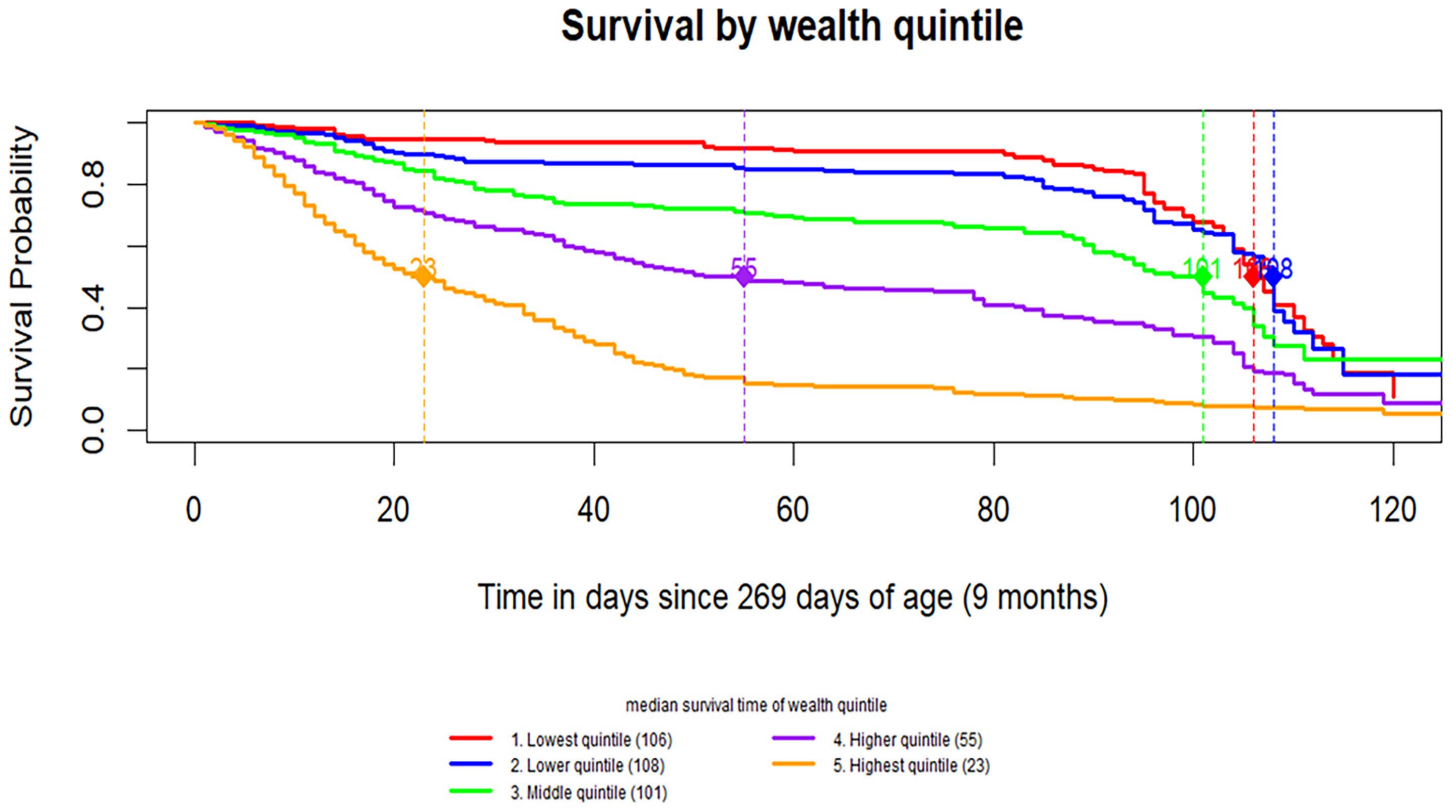
Wealth quantile survival curve of time to MCV1.

The survival probability varied across wealth quintile groups. The poorest groups showed noticeably higher survival probabilities compared to the wealthier groups. The median survival time was longest in the lower wealth quintiles (106 days for the lowest quintile and 108 days for the lower quintile) and progressively shorter in higher wealth groups, with the highest quintile showing the shortest median survival time, at 23 days (Figure 3).

### Multivariable cox proportional hazards model

The hazard of receiving MCV1 was 35% lower for pregnancies, which were not desired at all compared to pregnancies desired from the beginning. The hazard of receiving MCV1 was 21% lower among pregnancies that were initially undesired but later became wanted compared with those pregnancies that were desired from the beginning. The children from the highest wealth quintile had 4.35 times the hazard of MCV1 vaccination (95% CI: 3.10–6.11) compared with the lowest quintile. Protestant Christian (AHR = 0.63, 95% CI: 0.49–0.80) and Muslim (AHR = 0.60, 95% CI: 0.47–0.78) mothers were less likely to vaccinate their children timely than Orthodox Christian mothers. Institutional deliveries (hospitals/health centers) were associated with 62–68% timely vaccination uptake compared with home births (Table 3).

**Table 3:** Final multivariable model of time to first dose of measles-containing vaccine and associated factors.

Variable	Category	MCV1 status		CHR	AHR
		Event	Censor		
Religion	1. Orthodox	504 (28.47%)	167 (9.44%)	Ref.	Ref.
	2. Protestant	331 (18.7%)	234 (13.22%)	0.57 (0.42, 0.77)^***^	0.63 (0.49, 0.80)^***^
	3. Moslem/Muslim	320 (18.08%)	192 (10.85%)	0.54 (0.40, 0.75)^***^	0.60 (0.47, 0.78)^***^
	4. other	14 (0.79%)	8 (0.45%)	0.60 (0.32, 1.15)	1.04 (0.50, 2.15)
Wealth quintile	1. Lowest quintile	132 (7.46%)	150 (8.47%)	Ref.	Ref.
	2. Lower quintile	114 (6.44%)	157 (8.87%)	0.96 (0.73, 1.28)	0.96 (0.71, 1.28)
	3. Middle quintile	157 (8.87%)	127 (7.18%)	1.48 (1.05, 2.07)^*^	1.43 (1.03, 1.98)_*_
	4. Higher quintile	248 (14.01%)	91 (5.14%)	2.82 (1.92, 4.13)^***^	2.49 (1.66, 3.74)^***^
	5. Highest quintile	518 (29.27%)	76 (4.29%)	5.81 (4.24, 7.97)^***^	4.35 (3.10, 6.11)^***^
Last pregnancy desired	1. Then	826 (46.67%)	350 (19.77%)	Ref.	Ref.
	2. Later	294 (16.61%)	216 (12.2%)	0.71 (0.59, 0.85)^***^	0.79 (0.65, 0.96)^*^
	3. Not at all	49 (2.77%)	35 (1.98%)	0.68 (0.49, 0.95)^*^	0.65 (0.46, 0.93)^*^
Place of delivery	1. Home	225 (12.71%)	301 (17.01%)	Ref.	Ref.
	2. Government hospital	391 (22.09%)	107 (6.05%)	3.30 (2.63, 4.13)^***^	1.68 (1.30, 2.18)^***^
	3. Government health center	490 (27.68%)	183 (10.34%)	2.28 (1.86, 2.78)^***^	1.62 (1.30, 2.02)^***^
	4. Other	63 (3.56%)	10 (0.56%)	3.94 (2.29, 6.77)^***^	1.48 (0.91, 2.41)

AHR, adjusted hazard ratio, CHR, crude hazard ratio; CI, confidence interval; Ref, reference category, *** for *p*-values ≤ 0.001, ** for *p*-values ≤ 0.01, * for *p*-values ≤ 0.05.

## Discussion

The study included those children who were eligible for MCV1 at 9 months of age. The result showed that 27% of respondents received the MCV1 vaccination at the age of 9 to 10 months, indicating that more than two-third of the infants did not receive the MCV1 vaccination on time. Approximately 53.4% did not take MCV1 at 1 year of age, increasing the risk of the measles epidemic before complete immunity is achieved. This study’s findings were consistent with previous research findings conducted in the North Shoa zone of Ethiopia, and the Worabe data from EDHS (9, 40). The reason behind this low vaccination timing may be due to limited access to immunization services as a result of hard-to-reach areas, availability of MCV1 vaccines as a result of logistics barriers, such as vaccine carriers, fridge shortage, transportation, and misunderstandings about the ideal vaccination age in rural areas (21, 22, 41).

On the contrary, the findings of MCV1 timely vaccination rates were higher than previous study findings conducted in Ethiopia, such as the Somalia region, North Shoa, Oromia region, Gondar City, Wolaita Zone, and West Shewa Zone (39, 42–45). This PMA data is a national-level data that covers a large geographical region of the country, and it may include hard-to-reach areas for immunization that may not be included in these specific area studies. This may reflect differences in immunization access, differences in awareness about immunization benefits, and cultural attitudes toward vaccination in different areas of Ethiopia. The other inconsistency may be due to sample size and modeling differences; this study used national data and advanced statistical modeling (12, 22, 41).

In contrast, this study shows that MCV1 vaccination rates were lower in previous research findings from Ethiopian DHS data (46), suggesting that the interventions implemented in recent times may be more effective or better executed. This inconsistency may be explained by improved access to immunization services compared to previous years, strengthened community outreach programs, increased government investment in immunization, better coordination through health development armies, greater caregiver awareness of the benefits of timely vaccination, and improved communication about vaccine side effects (47, 48).

Institutional delivery was associated with timely receipt of MCV1. It is supported by previous study findings (24, 27, 39, 41, 49, 50). The reason behind this association is that maybe mothers who give birth at health facilities are more likely to receive comprehensive postnatal care, such as guidance on vaccination timelines and follow-up appointments. In addition, institutional delivery increases healthcare seeking behavior, which enhances adherence to recommended vaccination schedules (51–53).

Unintended pregnancy was associated with delayed receipt of MCV1. It is supported by previous studies (9, 54). Due to its potential impact on maternal and household dynamics, women who did not desire their pregnancies might experience reduced support from their partners or family members, which can affect their ability to access immunization services. This reduced support may also contribute to psychosocial stress and lower prioritization of immunization services, resulting in delays in taking their children to vaccinations. Furthermore, non-desired pregnancies might result in limited healthcare utilization during pregnancy and postpartum periods, leading to less awareness of vaccination schedules (55–58).

These findings suggest that children born to Protestant Christian and Muslim mothers were less likely to receive timely measles vaccination compared to those born to Orthodox Christian mothers. This disparity may reflect differences in beliefs toward vaccination, trust in the vaccination, or may reflect the belief that their faith protects children from disease (59, 60).

Children from wealthier households were associated with timely MCV1 uptake than those from poorer households. This association may be due to the role of socioeconomic inequality in access to timely vaccination. Wealthier families may have better access to health facilities for vaccination, higher health literacy, and fewer barriers to transportation or opportunity costs to immunization sites. In contrast, families in the lowest wealth quintile live in remote areas and may face transportation and lack of access to immunization sites, even when vaccines are provided free of charge (61–63).

### Strengths and limitations

This study has several strengths, such as a large, representative sample and the use of longitudinal data from the PMA Ethiopia cohort, which enhances validity. Advanced statistical methods using survey design survival analysis was used to control the collinearity of cluster effects, strata and survey weighting. The missing data was handled using multiple imputations. In addition, this study explicitly assessed the timeliness of MCV1 and its associated variables. However, this study did not consider the overall MCV1 coverage. The data were collected from four highly populated regions of the country, and other areas were not included, which may restrict generalizability to the country, Ethiopia. Moreover, the major limitation of the study is the lack of a comparison group, so that participants may have altered their behavior since similar questions were asked at each follow-up. This could affect the reliability of the findings. Potential unmeasured variables from this secondary dataset, such as maternal health beliefs, may influence vaccination behavior. There was a lack of convergence to the PNC counseling variable during multiple imputation since 71% of the data were missing, which may affect the validity of the variable significance.

## Conclusion

The findings showed that the timely MCV1 uptake was low. Place of delivery, maternal pregnancy intention, religion, and wealth quantiles were associated with the time to measles first dose. Infants born in health facilities were more likely to receive timely vaccinations. Unwanted pregnancies, poor wealth quantile, protestant and Muslim religion, and home of delivery were associated with delayed MCV1 uptake.

### Implications of the study

The findings of this study have several important implications for MCV1 uptake and immunization programs in Ethiopia. This study emphasizes the role of institutional deliveries in timely vaccination. Encouraging mothers to deliver in health facilities provides an opportunity to counsel them about timely vaccination and creates good caregiver and healthcare provider relationships. Health care providers are encouraged to integrate facility delivery with immunization, which in turn contributes to improved vaccination adherence.

Unwanted pregnancies lead to delayed vaccination. Healthcare providers, health system planners, and other stakeholders are advised to support unintended pregnancies, and educating mothers on the importance of vaccination and adhering to vaccination schedules can enhance timely immunization.

The health authorities need to collaborate more closely with religious leaders to build trust toward vaccination and tailor vaccine messaging to be respectful and relevant to religious beliefs. Implementing a continuous outreach immunization service helps low-income individuals obtain timely vaccination.

Future research are recommended to conducted longitudinal studies to assess how adherence to the MCV1 immunization contributes to sustained herd immunity and outbreak prevention.

## Data Availability

The data analyzed in this study is subject to the following licenses/restrictions: the data is accessible only when the objective of the study is stated to the performance monitoring for action (PMA) data set owners. Requests to access these datasets should be directed to https://www.pmadata.org/countries/ethiopia.
